# Juvenile Hormone (JH) Esterase of the Mosquito *Culex quinquefasciatus* Is Not a Target of the JH Analog Insecticide Methoprene

**DOI:** 10.1371/journal.pone.0028392

**Published:** 2011-12-09

**Authors:** Shizuo G. Kamita, Aman I. Samra, Jun-Yan Liu, Anthony J. Cornel, Bruce D. Hammock

**Affiliations:** 1 Department of Entomology, University of California Davis, Davis, California, United States of America; 2 University of California Davis Cancer Center, Sacramento, California, United States of America; Ghent University, Belgium

## Abstract

Juvenile hormones (JHs) are essential sesquiterpenes that control insect development and reproduction. JH analog (JHA) insecticides such as methoprene are compounds that mimic the structure and/or biological activity of JH. In this study we obtained a full-length cDNA, *cqjhe*, from the southern house mosquito *Culex quinquefasciatus* that encodes CqJHE, an esterase that selectively metabolizes JH. Unlike other recombinant esterases that have been identified from dipteran insects, CqJHE hydrolyzed JH with specificity constant (*k*_cat_/*K*_M_ ratio) and *V*_max_ values that are common among JH esterases (JHEs). CqJHE showed picomolar sensitivity to OTFP, a JHE-selective inhibitor, but more than 1000-fold lower sensitivity to DFP, a general esterase inhibitor. To our surprise, CqJHE did not metabolize the isopropyl ester of methoprene even when 25 pmol of methoprene was incubated with an amount of CqJHE that was sufficient to hydrolyze 7,200 pmol of JH to JH acid under the same assay conditions. In competition assays in which both JH and methoprene were available to CqJHE, methoprene did not show any inhibitory effects on the JH hydrolysis rate even when methoprene was present in the assay at a 10-fold higher concentration relative to JH. Our findings indicated that JHE is not a molecular target of methoprene. Our findings also do not support the hypothesis that methoprene functions in part by inhibiting the action of JHE.

## Introduction

**Figure 1:**
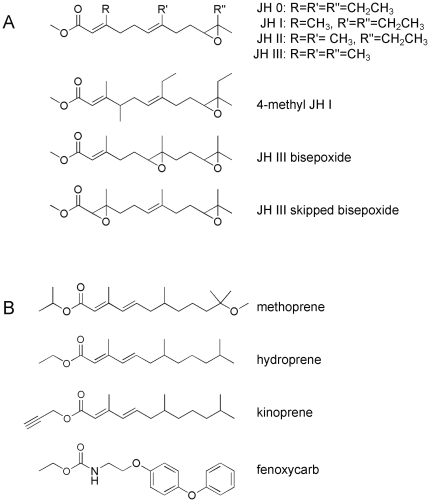
Chemical structures of juvenile hormone (JH) and JH analog (JHA) insecticides investigated in this study. Seven forms of JH have been isolated from insects; all posses a methyl ester at one end of the molecule and epoxide at the other (A). JH III is the principal form of JH that is found in dipteran insects. JHA insecticides (B) are structural and/or biological mimics of JH.

Juvenile hormones (JHs) are a group of structurally related sesquiterpenes that control a diversity of crucial life events in insects (reviewed in [1], [2]). JH was first identified as a hormone that regulates the type of molt that a juvenile insect will undergo. The presence of JH in the hemolymph at low nanomolar levels maintains the *status quo* so that a larva-to-larva or nymph-to-nymph molt occurs. In contrast, when JH levels precipitously fall from low nanomolar to picomolar levels and when there are concurrent spikes in ecdysteroid (molting hormone) levels, the insect undergoes a developmentally more advanced larva-to-pupa or nymph-to-adult molt. The precipitous reduction in JH titer results from a combination of the decreased biosynthesis of JH and increased metabolism of JH in the hemolymph and within cells [3]. Six major forms of JH (JH 0, JH I, JH II, JH III, 4-methy JH I, and JH III bisepoxide) have been isolated from insects; all possess an *α,β*-unsaturated methyl ester at one end of the molecule and an epoxide at the other (Fig. 1A). In addition JH III skipped bisepoxide, a novel JH in which the *α,β*-double bond is replaced by an epoxide, has been identified in a heteropteran insect [4].

The methyl ester of JH is metabolized by a JH-specific esterase (JHE) and the epoxide by a JH epoxide hydrolase (JHEH) [3]. Both JHE and JHEH belong to the α/β-hydrolase fold superfamily. JHEs show unique biochemical, structural, and biological characteristics that help to differentiate it from non-JH-specific esterases that are found in the hemolymph (reviewed in [5]). A hallmark of JHE is a specificity constant (*k*_cat_/*K*_M_) for JH that is generally greater than 10^6^ M^−1^ s^−1^. The driver for this high specificity constant is an exceptionally low apparent Michaelis constant (i.e., a *K*_M_ that is generally in the low nM range). On the other hand, the turnover of JH is slow with most JHEs showing a *k*_cat_ that is generally less than 2 s^−1^. JHEs show low nM-level sensitivity to 3-octylthio-1,1,1-trifluoropropan-2-one (OTFP), a slow tight binding inhibitor of esterases [6]. In contrast, the general esterase/serine protease inhibitor diisopropyl fluorophosphate (DFP) shows relatively poor inhibition of JHEs [7]. At the primary amino acid sequence level JHEs possess seven highly conserved sequence motifs (RF, DQ, GQSAG, E, GxxHxxD, R/Kx_(6)_R/KxxxR, and T) [5], [8]. Three of these motifs (GQSAG, E, and GxxHxxD) form the catalytic triad (catalytic residues are underlined).

Juvenile hormone analog insecticides (JHAs) such as methoprene, hydroprene, kinoprene, and fenoxycarb (Fig. 1B) are green compounds that mimic the chemical structure and/or biological action of JH (reviewed in [9], [10]). JHAs selectively target and disrupt the endocrine system of insects. When juvenile insects are exposed to JHAs at a time during development when JH titer is normally undetectable, abnormal larval-pupal or nymphal-pupal development and/or death is induced. JHAs are particularly suited for the control of pest and disease vectoring insects such as mosquitoes that have larval or nymphal stages that do not require fast kill (because they are innocuous) and/or commonly found in concentrated populations. JHAs can selectively target insects within an order or even at the family level; a level of selectively that is seldom obtained with more classical chemical insecticides. High target selectivity is one reason that JHAs are considered exceptionally safe. This safety is illustrated by the World Health Organization's acknowledgment of an acceptable methoprene use level of 1 mg liter^−1^ (i.e., 1 ppm) as a mosquito larvicide in human drinking water [11]. One caution though is that the acid metabolite of methoprene binds to the mammalian retinoid X receptor (RXR) alpha forming a transcriptional activator complex that is functional in mammalian cells [12]. The competitive displacement of the endogenous RXR ligand by methoprene acid, however, only occurs when it is present at relatively high concentrations.

Tolerance to methoprene, although uncommon, has been demonstrated in field populations of mosquitoes in Florida [13], California [14], and New York [15], and in the laboratory [16]. In the fruit fly, the absence or mutation of a so-called *methoprene-tolerant* (*met*) gene results in methoprene tolerance [17], [18]. The protein (MET) encoded by the *met* gene is a basic helix-loop-helix (bHLH)-PAS family transcriptional regulator protein [17] that binds JH with high affinity [19]. In the mosquito, an ortholog of MET is likely involved in JH binding and possibly methoprene tolerance following its mutation. Detoxification enzymes such as cytochrome P450, glutathione *S*-transferase, and/or carboxylesterase likely also play roles in tolerance to methoprene and other JHAs in dipteran insects [10], [20]. However, synergists of oxidases and carboxylesterases show relatively poor efficacy in bioassays with methoprene resistant *Ochlerotatus nigromaculis* suggesting that other factors such as poor penetration efficiency are involved in methoprene tolerance in this mosquito species [14].

Here we obtained a full-length, JHE-encoding cDNA (*cqjhe*) from the southern house mosquito *Cx. quinquefasciatus*, a geographically diverse and widespread mosquito whose genome has recently been sequenced [21]. The recombinant protein (CqJHE) encoded by *cqjhe* hydrolyzed JH with specificity constant (*k*_cat_/*K*_M_ ratio) and *V*_max_ values that are typical of known JHEs. CqJHE, however, showed no metabolism of methoprene (25 pmol in the assay) under assay conditions that were sufficient to produce 7,200 pmol of JH acid from JH. In competition assays in which both methoprene and JH were available to CqJHE, methoprene did not show any inhibitory effects on the rate of JH hydrolysis. Our findings indicate that JHE is not a molecular target of methoprene as has been previously hypothesized.

## Results

### Susceptibility of *Cx. quinquefasciatus* CQ1 to methoprene

**Figure 2:**
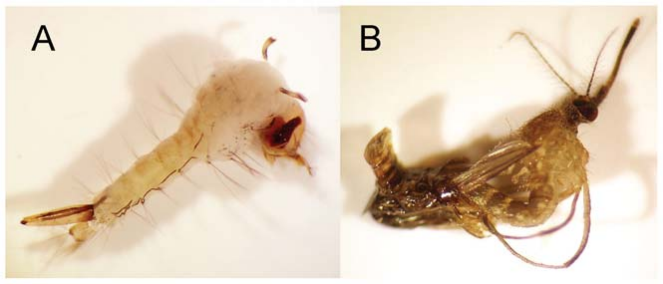
Effects of the juvenile hormone (JH) analog insecticide methoprene on mosquito development. Under normal conditions 4^th^ instar larval mosquitoes undergo a larval-pupal molt following a rapid reduction in hemolymph JH levels and concurrent spikes in molting hormone. When 4^th^ instar *Cx. quinquefasciatus* are exposed to methoprene at exceptionally low levels (60 to 120 ng liter^−1^) unique morphologies are observed including larval-pupal monsters (A) and insects that are unable to complete pupal-adult eclosion (B).

The mosquito strain *Cx. quinquefasciatus* CQ1 showed exceptional susceptibility to methoprene. Bioassays with methoprene on 4^th^ instar CQ1 gave a median lethal concentration (LC_50_) of 5.3 ng liter^−1^ (95% confidence limits of 0.01 to 39.8 ng liter^−1^) and LC_90_ of 4.4 µg liter^−1^ (95% confidence limits of 1.0 to 278 µg liter^−1^). Fourth instar larvae that were exposed to 60 to 120 ng liter^−1^ of methoprene typically died beginning on the 4^th^ day post exposure, and displayed morphology such as larval-pupal monsters (Fig. 2A) and incomplete adult eclosion (Fig. 2B) that are typically found in juvenile insects that are exposed to JHAs.

### Cloning and analysis of a JHE-encoding cDNA from *Cx. Quinquefasciatus*

**Figure 3:**
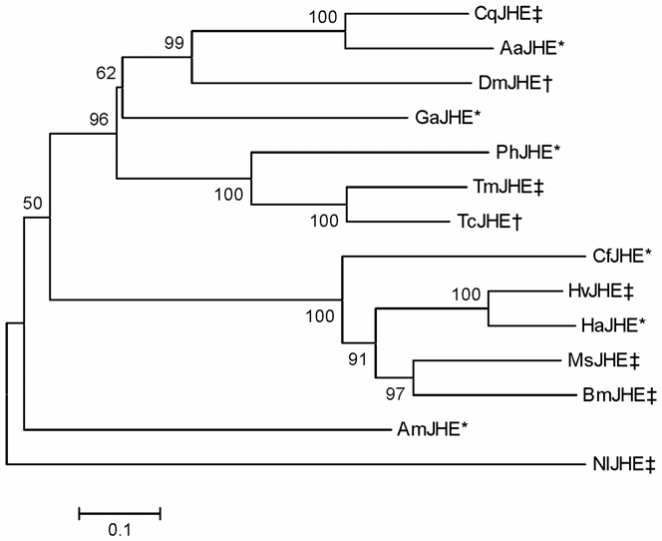
Phylogenetic relatedness of CqJHE and JHE sequences from six insect orders. The phylogenetic analysis was performed using MEGA version 5.05 [49]. The tree was generated by the Neighbor-Joining method using a ClustalW generated alignment of 14 JHE or putative JHE sequences. The percentage of replicate trees in which the sequences clustered together in the bootstrap analysis (1000 replicates) is shown at the branch nodes. The tree is drawn to scale, with branch lengths in the same units as those of the evolutionary distances (computed using the Poisson correction method) used to infer the phylogenetic tree. The double dagger (‡) and dagger (†) indicate proteins that show a specificity constant (*k*_cat_/*K*_M_) for JH III that is greater than or less than 10^6^ M^−1^ s^−1^, respectively. The asterisk (*) indicates that the specificity constant of the protein is uncharacterized. The insect order, GenBank accession number, and key reference of the sequences are as follows. Diptera: CqJHE (JN251105), AaJHE (EAT43357) [23], DmJHE (AF304352) [24]; Orthoptera: GaJHE (EF558769) [31]; Coleoptera: PhJHE (AB259898) [50], TmJHE (AF448479) [51], TcJHE (NP_001180223) [30]; Lepidoptera: CfJHE (AF153367) [52], HvJHE (AF037197) [53], HaJHE (FJ997319) [54], MsJHE (AF327882) [55], BmJHE (AF287267) [56]; Hymenoptera: AmJHE (AY647436) [57]; and Hemiptera: NlJHE (EU380769) [58].

The full-length, JHE-encoding cDNA of *Cx. quinquefasciatus* (i.e., *cqjhe*) was obtained by 3′-RACE with a degenerate primer that targeted the conserved GQSAG motif of JHE, and subsequently 5′-RACE with a gene-specific primer (Information S1). This approach identified a 2,019 nts-long cDNA (GenBank accession number JN251105) that contained an open reading frame of 1746 nts (Fig. S1). The 5′ and 3′ UTR sequences were 51 and 222 nts long, respectively. A 19 amino acid residues-long signal peptide for secretion was predicted at the N-terminal of the deduced protein (i.e., CqJHE) of *cqjhe* by SignalP 3.0 software [22]. The calculated mass of CqJHE lacking this putative signal peptide (563 amino acid residues) was 63,430 Daltons. CqJHE lacking its putative signal peptide had a computed pI of 5.64. CqJHE showed 96.6% identity to a putative JHE sequence (XM_001843289) found in the recently sequenced [21] JHB strain of *Cx. quinquefasciatus*. Interestingly, the C-terminal 18 amino acid residues of CqJHE showed no apparent homology with the corresponding region of the putative JHE of the JHB strain of *Cx. quinquefasciatus*. Phylogenetic analysis placed CqJHE in a clade that was clearly distinct from the well-studied lepidopteran JHEs (Fig. 3).

The seven amino acid sequence motifs (three catalytic and four non-catalytic) that are predictive of JHE were highly conserved in CqJHE (Fig. S1). The three predicted catalytic motifs (Fig. S1) were GQSAG, GVVHCDE, and D (predicted catalytic residues are underlined). Interestingly, the acidic residues in the conserved GxxHxxD and E motifs were substituted with E and D, respectively, in CqJHE. This is also the case in the putative JHE of the mosquito *Aedes aegypti* [23] but not in the JHE of the fruit fly [24]. The four non-catalytic JHE motifs were also conserved except for the amphipathic alpha helix motif (R/Kx_(6)_R/KxxxR) in which the last arginine residue was substituted with another basic residue histidine (Fig. S1). The amphipathic alpha helix motif is found near the surface of JHE and implicated in JHE degradation [25].

### Expression and purification of recombinant CqJHE

In order to test if *cqjhe* encoded a biologically active JHE, a recombinant baculovirus, AcCqJHE, carrying *cqjhe* was generated. AcCqJHE produced approximately 26 mg of CqJHE per liter of cell culture medium (containing approximately 2×10^6^ cells ml^−1^) of insect High Five cells. Approximately 94% of the JH hydrolytic activity was found in the cell culture supernatant at 65 h postinfection (Information S1). After the cell culture supernatant was diluted (1∶4) with 20 mM Tris-HCl, pH 8.0, and loaded onto a strong anion exchange column, it appeared that all of the detectable JH-specific esterase activity was bound. Roughly 20% and 60% of the JH-specific esterase activity was eluted from the anion exchange column in the 100 and 150 mM NaCl fractions (Table S1). After the 150 mM NaCl fraction was desalted and concentrated using a 30,000 NMWL Centriprep Ultracel YM-30 (Millipore) centrifugal filter device, CqJHE represented approximately 48% of the total proteins in this preparation (Fig. S2B). The specific activity of this preparation for JH III was 1,125 nmoles of JH III acid formed min^−1^ mg^−1^ of CqJHE. This represented a 19-fold increase in specific activity in comparison to that (57.8 nmoles min^−1^ mg^−1^) found in the supernatant of High Five cells infected with AcCqJHE. This purification factor was the same or better than that previously obtained for the well-characterized JHE of the tobacco hornworm *Manduca sexta* under the same purification scheme [26]. The 150 mM NaCl fraction, following desalting, was used as the enzyme source for all of the enzymatic activity analyses.

### Enzyme activity and kinetic analysis of CqJHE

CqJHE hydrolyzed the general esterase substrates *ρ*-nitrophenyl acetate (*ρ*-NPA) and *α*-naphthyl acetate (*α*-NA) with maximum velocities (*V*_max_) of 120 and 20 µmoles min^−1^ mg^−1^, respectively (Table 1). These velocities, however, required relatively high substrate concentrations in particular for *α*-NA as indicated by apparent Michaelis constants (*K*_M_) of 2,960 and 340 µM for *α*-NA and *ρ*-NPA, respectively (Table 1). The *V*_max_ of CqJHE for JH III was 1,200±40 nanomoles of JH III acid formed min^−1^ mg^−1^ when determined in 50 mM sodium phosphate buffer at pH 7.4. Although this rate was 1 to 2 orders of magnitude lower than those for the general esterase substrates, this rate was obtained at very low JH III concentrations (i.e., *K*_M_ of 130±19 nM). This exceptionally low *K*_M_ resulted in a specificity constant (*k*_cat_/*K*_M_) for JH III that was 26- and 1,400-fold higher than the general esterase substrates *ρ*-NPA and *α*-NA, respectively (Table 1). The significantly higher *V*_max_ values that CqJHE showed for *ρ*-NPA and *α*-NA (in comparison to JH III) likely results from the fact that the leaving groups of these general esterase substrates have significantly higher δ values resulting in the nitrophenol and naphthol being far better leaving groups than methanol. The specific activity of CqJHE for JH III was roughly 3-fold higher under alkaline pH conditions than under acidic pH condition (Fig. S3). Similarly, the *V*_max_ of CqJHE for JH III was 1.4-fold higher when it was determined in buffer at pH 9 than in buffer at pH 7.4 (Table 1). Like other JHEs, CqJHE showed exceptional stability when stored at 5°C (total protein concentration of 4.3 mg ml^−1^) with no detectable loss of JH hydrolytic activity after 12 months.

**Table 1:** Kinetic properties of CqJHE for *ρ*-nitrophenyl acetate, *α*-naphthyl acetate, and JH III^a^.

Substrate	*V*_max_ (µmol min^−1^ mg^−1^)	*K*_M_ (µM)	*k*_cat_ (s^−1^)	*k*_cat_/*K*_M_ (M^−1^ s^−1^)
*ρ*-nitrophenyl acetate^b^	120±2.0	340±17	130	3.8×10^5^
*α*-naphthyl acetate^c^	20±1.7	2960±420	21	7.1×10^3^
juvenile hormone III^b^	1.2±0.04	0.13±0.02	1.3	1.0×10^7^
juvenile hormone III^c^	1.7±0.03	0.13±0.01	1.8	1.4×10^7^

a The enzyme was purified by ion exchange chromatography as described in the text. The values given assume that the purity of the enzyme preparation (i.e., the 150 mM NaCl fraction after ion exchange) was 48%, and were corrected for background hydrolysis. The results shown are the mean ± standard deviation of at least three separate experiments.

b The assays were performed in 50 mM sodium phosphate buffer, pH 7.4, at 30°C.

c The assays were performed in glycine-sodium hydroxide buffer, pH 9.0, at 30°C.

### Inhibition of CqJHE by DFP or OTFP

Diisopropylfluorophosphate (DFP) is a potent inhibitor of serine proteases and a wide range of carboxylesterases. JHEs, however, are relatively resistant to the inhibitory effects of DFP. The IC_50_ of DFP for CqJHE was 634±54 nM. In contrast, the IC_50_ of OTFP for CqJHE was 0.23±0.05 nM. CqJHE was roughly 2,800-fold more sensitive to inhibition by OTFP than DFP.

### Interaction of methoprene and other JHAs with CqJHE

**Figure 4:**
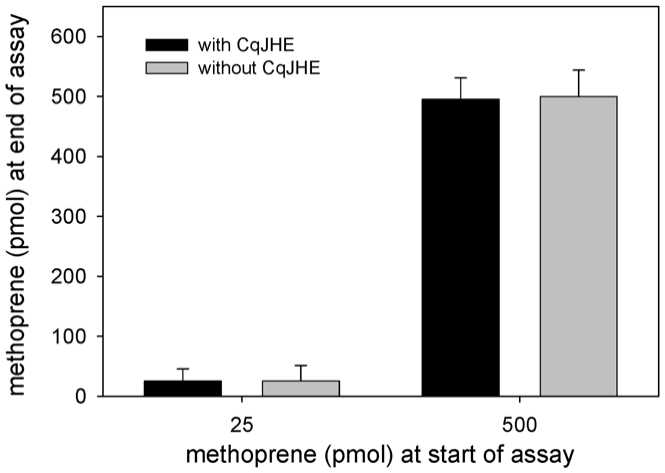
LC/MS/MS analysis of the metabolism of methoprene by CqJHE. In these assays 25 or 500 pmol of methoprene was incubated with 0.4 µg (6.3 pmol) of CqJHE (an amount that was sufficient to form 7,200 pmol of JH III acid under the same incubation conditions) for 15 min at 30°C. At the end of the incubation period, the reaction was stopped by the addition of methanol, and the amount of methoprene remaining was analyzed by LC/MS/MS. CUDA (12-(3-cyclohexylureido)dodecanoic acid) was used as an internal standard for LC/MS/MS. The error bars indicate the standard deviation of the mean of three independent experiments. No metabolism of methoprene was detected under the conditions tested.

CqJHE displayed all of the enzyme kinetic characteristics that are common to known JHEs indicating that CqJHE is a biologically significant in the metabolism of JH. CqJHE was thus used as a model dipteran JHE to ask the question of whether or not JHE is a target of methoprene and whether or not methoprene is metabolized by JHE. The extensive structural similarity between methoprene and JH (Fig. 1) suggested that methoprene would fit into the substrate-binding pocket of CqJHE and interact with the catalytic residues in a manner similar to JH. Analyses by LC/MS/MS following incubation of CqJHE with methoprene indicated that CqJHE does not metabolize methoprene. Specifically, when an amount of CqJHE (0.4 µg) that was sufficient to metabolize 7,200 pmol of JH III to JH III acid was incubated with 25 or 500 pmol of methoprene for 15 min at 30°C, no change was found in the amount of methoprene at the end of the assay (Fig. 4).

**Figure 5:**
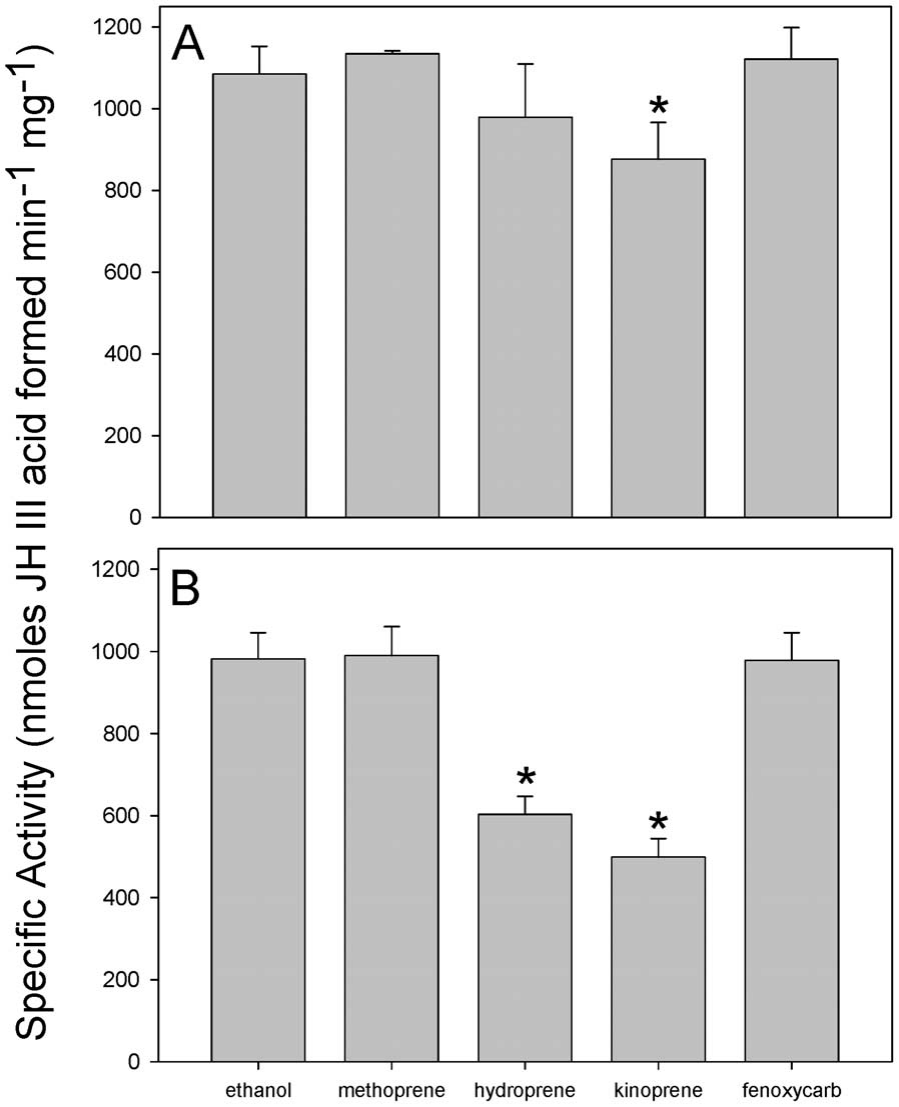
Effect of methoprene and other JHAs on the specific activity of CqJHE. The ability of methoprene and other JHAs to compete with JH III for the substrate binding pocket of CqJHE was determined in a reaction containing 2 ng (i.e., 32 fmol) of CqJHE and 5000 pmol of JH III or JHA (A) or 0.32 ng (i.e., 5.3 fmol) of CqJHE and 500 pmol of JH III and 5000 pmol of JHA (B). The error bars indicate the standard deviation of the mean of three independent experiments. Significant differences (*P*<0.001) in CqJHE specific activity between control reactions containing ethanol and experimental reactions containing a JHA are indicated by the asterisk (*).

The LC/MS/MS analyses clearly indicated that CqJHE does not metabolize methoprene, so we next asked if methoprene competes with JH for the binding pocket of CqJHE. In our initial competition experiments, CqJHE was incubated with JH III and methoprene at a molar ratio of 1∶1 (i.e., molar ratio of CqJHE∶JH III∶methoprene of 1∶15,600∶15,600). Under this condition, the presence of methoprene had no effect on the hydrolysis of JH III by CqJHE (Fig. 5A). In a subsequent experiment the molar amount of methoprene was increased 10-fold such that the molar ratio of CqJHE∶JH III∶methoprene was 1∶9,750∶94,300. The presence of methoprene at a 10-fold higher molar ratio in comparison to JH III also showed no effect on the hydrolysis of JH III by CqJHE (Fig. 5B). To our surprise, these findings indicated that methoprene does not interact with the substrate-binding pocket of CqJHE under the conditions of our assays. In contrast, two other commercially utilized JHAs hydroprene and kinoprene that share a similar chemical backbone as methoprene (Fig. 1B) were able to retard the hydrolysis of JH III by CqJHE (Fig. 5). This inhibition was most evident in the presence of a 10-fold higher molar ratio of hydroprene (40% inhibition) or kinoprene (50% inhibition) (Fig. 5). As was found with methoprene, fenoxycarb had no effect on the hydrolysis of JH III by CqJHE even when it was added to the reaction mixture at a 10-fold higher molar ratio in comparison to JH III (Fig. 5).

## Discussion

Mosquitoes in the genus *Culex* (primarily *Cx*. *pipiens* and *Cx*. *quinquefasciatus*) are major disease vectors that are found in nearly all regions of the world [27]. *Culex* complex mosquitoes transmit numerous human and veterinary pathogens including Rift Valley fever virus, St. Louis encephalitis virus, West Nile virus, *Wuchereria bancrofti*, avian malaria, and dog heartworm. Our ability to reduce the incidence of these diseases commonly involves controlling mosquito populations by reducing habitat sources and by applying chemical and biological insecticides. The availability of effective and safe insecticides is thus an integral component of mosquito control strategies. JHAs such as methoprene are effective and safe insecticides that disrupt the JH-based regulatory system of insects resulting in dramatic alterations in development and/or death.

The current dogma regarding the primary mode of action of methoprene and other JHAs is that they function as agonists of the JH receptor (reviewed in [9]). It has also been proposed that methoprene and other JHAs function as inhibitors of JHE and/or JHEH [28]. The inhibition of JHE and/or JHEH will putatively result in endogenous JH titers that do not fall below a critical cutoff level that is required for normal insect development to progress. In larval lepidopterans, the topical application of known inhibitors of JHE such as *O*-ethyl-*S*-phenyl phosphoramidothiolate (EPPAT) or OTFP induces pro-JH effects including delayed pupation, continued feeding, malformation, and giant larvae [7], [29]. In this study we tested the hypothesis that methoprene functions by inhibiting the enzymatic action of JHE. In order to generate a model system to test this hypothesis, *cqjhe* was cloned from 4^th^ instar *Cx. quinquefasciatus* and the protein encoded by *cqjhe* was expressed and characterized. The cloning of *cqjhe* and characterization of CqJHE was a critical part of our study since a recombinant dipteran esterase showing enzyme kinetic characteristics consistent with a JH-selective esterase was unavailable.

Genomic sequence analysis indicates that the dipteran and lepidopteran genomes potentially encode multiple *jhe* genes. Functional analysis of the proteins encoded by these potential *jhe* genes, however, indicates that each genome likely encodes only a single physiologically active JHE. For example, three potential *jhe* genes are found in *Ae. aegypti* [23] and five potential *jhe* genes are found in the silkworm *Bombyx mori* [30], however, only one of these sequences in each organisms appears to encode a physiologically functional JHE. Similarly, the genome of *Drosophila melanogaster* encodes four esterases with JHE-like characteristics, however, only one of these appears to be a physiologically functional JHE [31]. Screening of the recently determined genome sequence of *Cx*. *quinquefasciatus* (Johannesburg strain) [21] in VectorBase [32] identified 36 putative esterase genes of which 8 (CPIJ002073, CPIJ017763, CPIJ007135, CPIJ007424, CPIJ018753, CPIJ018752, CPIJ004066, and CPIJ013175) were described as “juvenile hormone esterase” or “juvenile hormone esterase precursor”. The JHE-encoding cDNA identified in this study (i.e., *cqjhe*) showed the highest identity (96.6%) to CPIJ002073 (GenBank accession number XM_001843289). The remaining 7 sequences generally showed less than 50% identity to *cqjhe*. We hypothesize that *cqjhe* is the only sequence in the genome of *Cx. quinquefasciatus* that encodes a biologically significant JHE, however, this hypothesis must still be experimentally tested.

Two recombinant esterases that are thought to be JHEs have been characterized from the dipterans *D. melanogaster* and *Ae. aegypti*. The recombinant esterase reported from *D. melanogaster* metabolizes JH III with a relatively high *K*_M_ (1,500 nM) resulting in a relatively low specificity constant (6.0×10^5^ M^−1^ s^−1^) for JH III [31]. In comparison, authentic JHE of *D. melanogaster* hydrolyzes JH III with a *K*_M_ of 89 nM resulting in a specificity constant that is about 10-fold higher [33]. The enzyme kinetic constants of the recombinant esterase reported from *Ae. aegypti* are undetermined [23]. The specific activity of this esterase (expressed by a recombinant baculovirus in Sf9 cells) for JH III is only 0.226 pmoles of JH acid formed min^−1^ mg^−1^ [23]. In comparison, the specific activity of CqJHE (57.8 nmoles min^−1^ mg^−1^) that was expressed in this study under similar conditions was about 250,000-fold higher. CqJHE is, thus, the first and only recombinant dipteran esterase to date that is known to possess primary sequence motifs and characterized enzyme kinetics that are completely consistent with a physiologically functional JHE (Fig. S1 and Table 1). The availability of a convenient source of a dipteran JHE thus allowed us to test the hypothesis that methoprene functions as an inhibitor of JHE.

The high structural similarity between methoprene and JH III (Fig. 1) suggests that methoprene is an ideal surrogate substrate of CqJHE. However, when 25 or 500 pmol of methoprene was incubated with CqJHE under assay conditions that were sufficient to hydrolyze 7,200 pmol of JH III to JH III acid, no hydrolysis of methoprene was observed. The inability of CqJHE to hydrolyze the isopropyl ester of methoprene was surprising but consistent with similar experiments using (1) homogenates prepared from larval northern house mosquito *Cx. pipiens pipiens* [34] and (2) dilute hemolymph from *M. sexta* [35] which show no apparent metabolism of methoprene. These findings and our findings may be partially explained by the structure of the substrate-binding pocket of JHE. In the case of the JHE of the lepidopteran *M. sexta* the catalytic triad is found at the end of an unusually long and narrow pit that is lined by hydrophobic amino acid residues [36]. The location of the catalytic triad within the substrate-binding pocket thus may not allow sufficient room for anything larger than a methyl ester to bind in the appropriate conformation for hydrolysis. A previous study from our laboratory with a series of surrogate thioester substrates also found that only methyl thioesters with long hydrophobic backbones are metabolized by a lepidopteran JHE [37].

Our results clearly indicated that methoprene is not a substrate of CqJHE so we next asked if the presence of methoprene inhibits the hydrolysis of JH. The presence of both methoprene and JH III in the JH hydrolysis assay did not affect the ability of CqJHE to metabolize JH III indicating that methoprene does not inhibit or otherwise interact with JHE as was previously hypothesized [28]. These findings are consistent with previous studies showing that methoprene is a poor inhibitor of authentic semi-purified JHEs and purified JHE (with JH III substrate) from multiple insect orders including Blattaria, Lepidoptera, Coleoptera, and Diptera [7], [33], [38]. In similar competition experiments (i.e., when both JH and the JHA are present at the same molar ratio), hydroprene and fenoxycarb (composed of ethyl esters) and kinoprene (composed of a propynyl ester) were also unable to inhibit or only poorly inhibited the hydrolysis of JH III (Fig. 5A). Hydroprene and kinoprene, however, were able to significantly inhibit the hydrolysis of JH III by CqJHE when present at a 10-fold higher molar ratio suggesting that these JHAs bind the substrate-binding pocket of CqJHE and prevent binding and hydrolysis of JH (Fig. 5B).

In conclusion, our findings indicate that methoprene is not metabolized by mosquito JHE and furthermore methoprene does not inhibit JH hydrolysis. Our findings do not contradict the current dogma that JHAs function primarily as agonists or partial agonists of the JH receptor. Our data also do not contradict hypotheses that methoprene and other JHAs may function by preventing the binding of JH to transcriptional regulatory proteins such as MET and/or JH binding proteins in the hemolymph or within cells. Given the large number of non-JH-specific esterases that are found in insects, methoprene and other JHAs may function in part as inhibitors of these non-JH-specific esterases.

## Materials and Methods

### Ethics statement

All work involving non-human primates was conducted according to relevant national and international guidelines in order to ameliorate suffering. The UC Davis Institutional Animal Care and Use Committee has approved all work involving non-human primates (animal care and use protocol #15953).

### JHAs, substrates, and inhibitors

The JHAs (methoprene, *S*-hydroprene, kinoprene, and fenoxycarb), general esterase substrates (*α*-naphthyl acetate (*α*-NA) and *ρ*-nitrophenyl acetate (*ρ*-NPA)), unlabeled JH III, and serine protease/esterase inhibitor diisopropyl fluorophosphate (DFP) were purchased from Sigma-Aldrich. Tritium-labeled JH III (11.5 Ci/mmol) was purchased from PerkinElmer. 3-Octylthio-1,1,1-trifluoropropan-2-one (OTFP), a slow and tight binding inhibitor of JHE [6] was synthesized in the laboratory as described previously [29]. 12-(3-Cyclohexylureido)dodecanoic acid (CUDA) was also synthesized in the laboratory as descried previously (compound #42 in [39]).

### Mosquito strain and rearing

The CQ1 strain of *Cx. quinquefasciatus* (formerly known as *Cx. pipiens quinquefasciatus*) was field-collected in Merced County, California, in the early 1950s [40] and subsequently maintained in the laboratory. The CQ1 strain has never been exposed to methoprene or other JHA. CQ1 larvae were reared in plastic pans (30×25 cm) containing 2 liters of tap water and fed on ground rodent diet (LabDiet 5001, PMI Nutrition International, Brentwood, MO). CQ1 adults were maintained on a 10% (w∶v) solution of sucrose in tap water. Blood feeding of adults prior to egg production was done on mice. The UC Davis Institutional Animal Care and Use Committee has approved the mouse blood feeding protocol under animal care and use protocol #15953. Larvae and adults were reared under a 12∶12 (light∶dark) photoperiod at 28°C.

### Larval Bioassay

Bioassays were performed with groups of 19 to 22 late 4^th^ instar larvae (i.e., larvae that were predicted to undergo larval-pupal molting within 24 h in the absence of methoprene treatment) as essentially described previously [14]. The bioassays were performed in 8 ounce glass jars (Qorpak, Bridgeville, PA) that were silanized with Sigmacote (Sigma-Aldrich). Following treatment with Sigmacote, the jars were rinsed with hot tap water, then dionized H_2_O, and then twice with acetone prior to baking at 80°C for at least 12 h. Each jar contained 100 ml of conditioned tap water (i.e., tap water that was left on the bench top for at least 12 h), methoprene (58.5 to 58,500 ng liter^−1^), 0.5% (v∶v) acetone, 5 mg of bovine liver powder (MP Biomedicals), and 19 to 22 larvae. Each jar was preincubated for at least 24 h with the same concentration of methoprene as was used in the assay. At least four replicates were performed for each methoprene concentration. The larvae were reared under a 12∶12 (light∶dark) photoperiod at 28°C. Mortality was recorded at 24 h intervals post treatment until the larvae died or underwent larval-adult eclosion. Median lethal concentration (LC_50_) and LC_90_ were determined using the POLO program [41].

### The JHE-encoding cDNA of *Cx. quinquefasciatus* and generation of AcCqJHE

The JHE-encoding cDNA of *Cx. quinquefasciatus* (*cqjhe*) was cloned from 4^th^ instar CQ1 larvae as described in detail in the Information S1. The coding sequence of *cqjhe* was inserted into the baculovirus transfer vector plasmid pAcUW21 as described in Information S1 and the resulting recombinant transfer vector pAcUW21-CqJHE was used to generate the recombinant baculovirus AcCqJHE. AcCqJHE was generated by transfecting Sf9 cells (Invitrogen) with 1.9 µg of pAcUW21-CqJHE and 1.6 µg *Bsu*36I-digested BacPAK6 baculovirus DNA (Clontech) using Cellfectin Transfection Reagent (Invitrogen) following the manufacturer's protocol. The Sf9 cells were cultured on ExCell 420 medium (SAFC Biosciences) supplemented with 2.5% fetal bovine serum at 27°C. AcCqJHE was isolated by three rounds of plaque purification on Sf9 cells following standard procedures [42].

### Expression and purification of CqJHE

CqJHE was expressed in High Five cells (Invitrogen) that were inoculated with AcCqJHE at a multiplicity of infection of 0.5 and cultured on ESF921 medium (Expression Systems) at 27°C. At 65 h p.i. the cell culture supernatant was harvested following centrifugation as described above, diluted 1∶4 with 20 mM Tris-HCl, pH 8.0, and applied onto a strong anion exchange spin column (Pierce). The spin column was washed once with 20 mM Tris-HCl, pH 8.0. The proteins were eluted with Tris-HCl, pH 8.0, containing increasing concentrations (100, 150, 200, and 300 mM) of NaCl. The majority of the activity was found in the 150 mM (60%) and 100 mM (22%) NaCl fractions (Table S1). These fractions were subjected to desalting and concentration using Centriprep YM-30 filtration devices (Millipore) and analyzed by sodium dodecyl sulfate-polyacrylamide gel electrophoresis (SDS-PAGE) (Fig. S2A). Protein concentrations were determined using a Bradford method-based protein assay reagent (Bio-Rad) using bovine serum albumin (BSA) fraction V (Sigma) to generate a standard curve. SDS-PAGE was performed using 10% NuPAGE Novex Bis-Tris gel (Invitrogen) using NuPAGE MOPS running buffer (Invitrogen). The gels were stained with Bio-Safe Coomassie stain (Bio-Rad) following the manufacturer's protocol. Molecular weight was estimated by comparison to SeeBlue Plus2 prestained standards (Invitrogen). The efficiency of the purification scheme for CqJHE was determined following SDS-PAGE separation of proteins using the ImageJ program [43]. The CqJHE-specific band was identified by treating the protein solution with MBTFP-Sepharose [44] to remove JHE or left untreated (Fig. S2B). The enzyme preparation from the 150 mM NaCl fraction was used as the source of enzyme for all of the CqJHE activity and inhibition analyses.

### Enzyme assays and kinetic constant determinations

The ability of CqJHE to hydrolyze the general esterase substrates *ρ*-NPA and *α*-NA was determined by spectrophotometric assays as described previously [45], [46]. The *ρ*-NPA assay was performed in a 200 µl reaction volume containing 40 ng of CqJHE, *ρ*-NPA (5 to 1,500 µM), 0.44% (v∶v) ethanol, and 0.1 mg ml^−1^ BSA in 50 mM sodium phosphate buffer, pH 7.4. The reaction was allowed to proceed for at 30°C for 5 min with measurements taken at 6 sec intervals at OD_405_. The *α*-NA assay was performed in a 297 µl reaction volume containing 40 ng CqJHE, *α*-NA (250 to 3,500 µM), 0.67% (v∶v) ethanol, and 0.1 mg ml^−1^ BSA in glycine-sodium hydroxide buffer, pH 9.0. The reaction was allowed to proceed at 30°C for 10 min with measurements taken at 16 sec intervals at OD_450_. Standard curves were generated under identical assay conditions using *ρ*-nitrophenol (Sigma-Aldrich) and *α*-naphthol (Sigma-Aldrich) in order to generate molar extinction coefficients for *ρ*-NPA and *α*-NA, respectively. Molar extinction coefficients of 6.80 OD_405_ mM^−1^ and 13.99 OD_450_ mM^−1^ were obtained for *ρ*-NPA and *α*-NA, respectively, under these conditions. The Michaelis constant (*K*_M_) and *V*_max_ were determined using the SigmaPlot Enzyme Kinetics Module 1.1 (Systat Software) with at least nine different concentrations of *ρ*-NPA or *α*-NA that bracketed the estimated *K*_M_ value. The *k*_cat_ of CqJHE for *ρ*-NPA and *α*-NA was calculated using an estimated molecular mass of 63.4 kDa. The assays were performed in quadruplicate and repeated four times.

The ability of CqJHE to hydrolyze JH III was determined by a partition assay as described previously [47]. The JH partition assay was routinely performed in a 100 µl reaction volume containing CqJHE, 5 µM JH III, 1% (v∶v) ethanol, and 0.1 mg ml^−1^ BSA in 50 mM sodium phosphate buffer, pH 7.4, at 30°C. *K*_M_ and *V*_max_ were determined using the SigmaPlot Enzyme Kinetics Module 1.1 software using specific activity data obtained with six different JH III concentrations (17.4 to 5,017 nM) that bracketed the estimated *K*_M_ value. The enzyme concentration and/or assay time (5 to 30 min) were adjusted in these assays so that at least 5% but no more than 15% of the JH III substrate was hydrolyzed. The *k*_cat_ of CqJHE for JH III was calculated using an estimated molecular mass of 63.4 kDa. All of the assays were performed in triplicate and repeated at least three times.

### Inhibition of CqJHE by DFP and OTFP

The abilities of the general serine protease/esterase inhibitor DFP and JHE inhibitor OTFP to inhibit CqJHE were determined by a modification of the JH partition assay described above. The inhibitor (diluted in ethanol) was preincubated with the enzyme for 15 min at 30°C prior to the addition of JH III. In these assays, the final ethanol concentration was 2% (v∶v). DFP containing assays were performed in 50 mM sodium phosphate buffer, pH 7.4, whereas OTFP containing assays were performed in glycine-sodium hydroxide buffer, pH 9.0. Six different concentrations (50 to 5,000 nM) of DFP and eight different concentrations (0.0024 to 400 nM) of OTFP were used to determine the half maximal inhibitory concentration (IC_50_) of these compounds. IC_50_s and were calculated using the Regression Wizard subprogram (Sigmoid, 4 Parameter equation) of SigmaPlot 2001 (Systat Software). The assays were performed in triplicate and repeated three times for each concentration of inhibitor.

### LC/MS/MS analysis of methoprene metabolism by CqJHE

The ability of CqJHE to metabolize methoprene was investigated by LC/MS/MS generally as described previously [48] except that a derivatization step to improve the limit of detection of methoprene was not performed. The assay was performed in a 100 µl reaction volume containing 0.4 µg (i.e., 6.3 pmol) of CqJHE (an amount that was sufficient to form 7,200 picomoles of JH III acid during the incubation period), 0.25 or 5 µM methoprene (i.e., 25 or 500 pmol, respectively), 2% (v∶v) ethanol, and 0.1 mg ml^−1^ of BSA in 50 mM sodium phosphate buffer, pH 7.4. After a 15 min-long incubation at 30°C the reaction was stopped by the addition of 200 µl of methanol, and then centrifuged at 1,790 ×*g* for 5 min at room temperature to precipitate protein. Subsequently, 100 µl of the supernatant was mixed with 100 µl of a 400 nM solution of CUDA (200 nM final concentration) which served as an internal standard for LC/MS/MS.

### Effect of JHAs on JH III hydrolytic activity of CqJHE

The ability of the JHAs methoprene, hydroprene, kinoprene, and fenoxycarb to compete with JH III for the binding pocket of CqJHE (or act as an inhibitor of CqJHE) was determined by a modification of the JH partition assay described above. The competition assay was performed in a 100 µl reaction volume so that the molar ratio of JH III and each JHA was 1∶1 or 1∶10. The “1∶1” reaction contained 2 ng (i.e., 32 fmol) of CqJHE, 5 µM (i.e., 500 pmol) JH III, 5 µM (i.e., 500 pmol) JHA, 2% (v∶v) ethanol, and 0.1 mg ml^−1^ of BSA in 50 mM sodium phosphate buffer, pH 7.4. For the “1∶10” reaction the JH III concentration was reduced to 0.5 µM (i.e., 50 pmol) and the amount of enzyme was reduced to 0.33 ng. The JH III (and CqJHE) concentration was decreased by 10-fold instead of increasing the amount of the JHA because 5 µM is near the solubility limit of the JHAs in water. In these assays, the JHA (diluted in ethanol) was added to the reaction mixture first then immediately afterwards the JH III was added, and the mixture was incubated at 30°C for 15 min. The assays were performed in triplicate and repeated three times. Statistically significant differences between the means of two treatments were determined by two-tailed Student's *t* tests.

## Supporting Information

Figure S1 **Nucleotide and deduced amino acid sequences of** ***cqjhe*** **and CqJHE.** The 5′ and 3′ UTR sequences, and coding sequence of *cqjhe* were 51, 222, and 1746 nts-long, respectively. Seven amino acid sequence motifs (RF, DQ, GQSAG, E, GxxHxxD/E, R/Kx_(6)_R/KxxxR, and T) are highly conserved in known JHEs [2], [3]. The RF (residues 74–75), DQ (residues 197–198), GQSAG (residues 223–227), D (residue 360), GxxHxxE (residues 480, 483, and 486), and T (residue 317) motifs are shown in bold underlined text. The E motif (i.e., the acidic amino acid residue of the catalytic triad) is D in CqJHE. The Kx_(6)_RxxxH motif (residues 196, 203, and 207) is shown in bold italic text. The asterisk indicates a stop codon (TAA). A putative signal peptide sequence (N-terminal 19 amino acid residues) is shown in italic text. A putative CPSF (cleavage and polyadenylation specificity factor) complex binding site is underlined. Amino acid residue positions are indicated to the right.
(TIF)

Figure S2 **SDS-PAGE analysis of CqJHE following ion exchange purification (A) and binding with MBTFP-Sepharose (B).** CqJHE was expressed in High Five cells by AcCqJHE and the culture supernatant (lane 1) was harvested at 65 h postinoculation, diluted (1∶4) with 20 mM Tris-HCl, pH 8.0, and applied onto a strong anion exchange column (Pierce). The column was washed and the proteins were eluted with 20 mM Tris-HCl, pH 8.0, containing increasing concentrations of NaCl. The majority of CqJHE activity eluted in buffer containing 100 (lane 2) or 150 (lane 3) mM NaCl. The protein solutions were desalted and concentrated, and the efficiency of the purification scheme was investigated by treating an equal volume (containing the same amount of total protein) of the 100 mM NaCl (lanes 4 and 5) or 150 mM NaCl (lanes 6 and 7) fractions with MBTFP-Sepharose (lanes 5 and 7), a JHE-selective affinity gel [4]. Treatment of the 100 mM and 150 mM NaCl fractions with MBTFP-Sepharose resulted in 94% and 95% reductions, respectively, in JHE specific activity. The masses (in kDa) of molecular weight standards (lane M) are indicated to the right of each panel. In panel A, 5 µg of total protein was separated in each lane; whereas in panel B, 7.5 µg of total protein was left untreated (lanes 4 and 6) or treated with MBTFP-Sepharose (lanes 5 and 7) prior to separation by SDS-PAGE.
(TIF)

Figure S3 **Effect of pH on the specific activity of CqJHE.** The partition assay (see Materials and Methods) was performed in citrate-phosphate (pH 4.0 and 5.0), sodium phosphate (pH 6.0, 7.0, and 8.0) or glycine-sodium hydroxide (pH 9.0 and 10.0) buffer containing 0.1 mg ml^−1^ of BSA. All of the assays were corrected for a low level of background hydrolysis (5.0±3.2%, 2.3±0.4%, 1.4±0.4%, 1.2±0.2%, 1.3±0.3%, 1.4±0.3%, 1.3±0.4% at pH 4, 5, 6, 7, 8, 9, and 10, respectively) that was found at each pH level. The error bars indicate the standard deviation of the mean of at least three independent experiments.
(TIF)

Information S1 **Materials and Methods.**
(DOC)

Table S1 **Purification of CqJHE by ion exchange chromatography.**
(DOC)

## References

[1] Goodman WG, Granger NA, Gilbert LI, Iatrou K, Gill SS (2005). The juvenile hormones.. Comprehensive Molecular Insect Science.

[2] Riddiford LM (2008). Juvenile hormone action: A 2007 perspective.. J Insect Physiol.

[3] Hammock BD, Kerkut GA, Gilbert LI (1985). Regulation of juvenile hormone titer: degradation.. Comprehensive Insect Physiology, Biochemistry, and Pharmacology.

[4] Kotaki T, Shinada T, Kaihara K, Ohfune Y, Numata H (2009). Structure determination of a new juvenile hormone from a heteropteran insect.. Org Lett.

[5] Kamita SG, Hammock BD (2010). Juvenile hormone esterase: biochemistry and structure.. J Pestic Sci.

[6] Abdel-Aal YAI, Hammock BD (1985). 3-Octylthio-1,1,1-trifluoro-2-propanone, a high affinity and slow binding inhibitor of juvenile hormone esterase from *Trichoplusia ni* (Hubner).. Insect Biochem.

[7] Sparks TC, Hammock BD (1980). Comparative inhibition of the juvenile hormone esterases from *Trichoplusia ni*, *Tenebrio molitor*, and *Musca domestica*.. Pestic Biochem Physiol.

[8] Ward VK, Bonning BC, Huang T, Shiotsuki T, Griffeth VN (1992). Analysis of the catalytic mechanism of juvenile hormone esterase by site-directed mutagenesis.. Int J Biochem.

[9] Wilson TG (2004). The molecular site of action of juvenile hormone and juvenile hormone insecticides during metamorphosis: how these compounds kill insects.. J Insect Physiol.

[10] Henrick CA (2007). Methoprene.. J Am Mosq Control Assoc.

[11] Cotruvo J, Fawell JK, Giddings M, Jackson P, Magara Y (2008). Methoprene in drinking-water: Use for vector control in drinking-water sources and containers.

[12] Harmon MA, Boehm MF, Heyman RA, Mangelsdorf DJ (1995). Activation of mammalian retinoid-X receptors by the insect growth regulator methoprene.. Proc Natl Acad Sci USA.

[13] Dame DA, Wichterman GJ, Hornby JA (1998). Mosquito (*Aedes taeniorhynchus*) resistance to methorprene in an isolated habitat.. J Am Mosq Control Assoc.

[14] Cornel AJ, Stanich MA, McAbee RD, Mulligan FS (2002). High level methoprene resistance in the mosquito *Ochlerotatus nigromaculis* (Ludlow) in Central California.. Pest Manag Sci.

[15] Paul A, Harrington LC, Zhang L, Scott JG (2005). Insecticide resistance in *Culex pipiens* from New York.. J Am Mosq Control Assoc.

[16] Brown TM, Brown AWA (1974). Experimental induction of resistance to a juvenile hormone mimic.. J Econ Entomol.

[17] Ashok M, Turner C, Wilson TG (1998). Insect juvenile hormone resistance gene homology with the bHLH-PAS family of transcriptional regulators.. Proc Natl Acad Sci USA.

[18] Wilson TG, Ashok M (1998). Insecticide resistance resulting from an absence of target-site gene product.. Proc Natl Acad Sci USA.

[19] Miura K, Oda M, Makita S, Chinzei Y (2005). Characterization of the *Drosophila Methoprene*-tolerant gene product - Juvenile hormone binding and ligand-dependent gene regulation.. FEBS J.

[20] Hemingway J, Hawkes NJ, McCarroll L, Ranson H (2004). The molecular basis of insecticide resistance in mosquitoes.. Insect Biochem Mol Biol.

[21] Arensburger P, Megy K, Waterhouse RM, Abrudan J, Amedeo P (2010). Sequencing of *Culex quinquefasciatus* establishes a platform for mosquito comparative genomics.. Science.

[22] Bendtsen JD, Nielsen H, von Heijne G, Brunak S (2004). Improved prediction of signal peptides: SignalP 3.0.. J Mol Biol.

[23] Bai H, Ramaseshadri P, Palli SR (2007). Identification and characterization of juvenile hormone esterase gene from the yellow fever mosquito, *Aedes aegypti*.. Insect Biochem Mol Biol.

[24] Campbell PM, Harcourt RL, Crone EJ, Claudianos C, Hammock BD (2001). Identification of a juvenile hormone esterase gene by matching its peptide mass fingerprint with a sequence from the *Drosophila* genome project.. Insect Biochem Mol Biol.

[25] Thomas BA, Church WB, Lane TR, Hammock BD (1999). Homology model of juvenile hormone esterase from the crop pest, *Heliothis virescens*.. Proteins: Struct, Funct, Genet.

[26] Hinton AC, Hammock BD (2003). In vitro expression and biochemical characterization of juvenile hormone esterase from *Manduca sexta*.. Insect Biochem Mol Biol.

[27] Vinogradova EB (2000). *Culex pipiens pipiens* mosquitoes: taxonomy, distribution, ecology, physiology, genetics, applied importance and control.

[28] Slade M, Wilkinson CF (1973). Juvenile hormone analogs: A possible case of mistaken identity?. Science.

[29] Hammock BD, Abdel-Aal YAI, Mullin CA, Hanzlik TN, Roe RM (1984). Substituted thiotrifluoropropanones as potent selective inhibitors of juvenile hormone esterase.. Pestic Biochem Physiol.

[30] Tsubota T, Shimomura M, Ogura T, Seino A, Nakakura T (2010). Molecular characterization and functional analysis of novel carboxyl/cholinesterases with GQSAG motif in the silkworm *Bombyx mori*.. Insect Biochem Mol Biol.

[31] Crone EJ, Sutherland TD, Campbell PM, Coppin CW, Russell RJ (2007). Only one esterase of *Drosophila melanogaster* is likely to degrade juvenile hormone *in vivo*.. Insect Biochem Mol Biol.

[32] Lawson D, Arensburger P, Atkinson P, Besansky NJ, Bruggner RV (2007). VectorBase: a home for invertebrate vectors of human pathogens.. Nucleic Acids Res.

[33] Campbell PM, Oakeshott JG, Healy MJ (1998). Purification and kinetic characterisation of juvenile hormone esterase from *Drosophila melanogaster*.. Insect Biochem Mol Biol.

[34] Brown TM, Hooper GHS (1979). Metabolic detoxication as a mechanism of methoprene resistance in *Culex pipiens pipiens*.. Pestic Biochem Physiol.

[35] Weirich G, Wren J (1973). The substrate specificity of juvenile hormone esterase from *Manduca sexta* haemolymph.. Life Sci.

[36] Wogulis M, Wheelock CE, Kamita SG, Hinton AC, Whetstone PA (2006). Structural studies of a potent insect maturation inhibitor bound to the juvenile hormone esterase of *Manduca sexta*.. Biochemistry.

[37] McCutchen BF, Uematsu T, Székács A, Huang TL, Shiotsuki T (1993). Development of surrogate substrates for juvenile hormone esterase.. Arch Biochem Biophys.

[38] Hammock BD, Sparks TC, Mumby SM (1977). Selective inhibition of JH esterases from cockroach hemolymph.. Pestic Biochem Physiol.

[39] Morisseau C, Goodrow MH, Newman JW, Wheelock CE, Dowdy DL (2002). Structural refinement of inhibitors of urea-based soluble epoxide hydrolases.. Biochem Pharmacol.

[40] McAbee RD, Kang KD, Stanich MA, Christiansen JA, Wheelock CE (2004). Pyrethroid tolerance in *Culex pipiens pipiens* var *molestus* from Marin County, California.. Pest Manag Sci.

[41] Russell RM, Robertson JL, Savin NE (1977). POLO: a new computer program for probit analysis.. Entomol Soc Am Bull.

[42] Merrington CL, King LA, Posse RD, Higgins SJ, Hames BD (1999). Baculovirus expression systems..

[43] Rasband WS (2006). ImageJ.

[44] Abdel-Aal YAI, Hammock BD (1986). Transition state analogs as ligands for affinity purification of juvenile hormone esterase.. Science.

[45] Mastropaolo W, Yourno J (1981). An ultraviolet spectrophotometric assay for α-naphthyl acetate and α-naphthyl butyrate esterases.. Anal Biochem.

[46] Kamita SG, Hinton AC, Wheelock CE, Wogulis MD, Wilson DK (2003). Juvenile hormone (JH) esterase: Why are you so JH specific?. Insect Biochem Mol Biol.

[47] Hammock BD, Sparks TC (1977). A rapid assay for insect juvenile hormone esterase activity.. Anal Biochem.

[48] Aronov PA, Dettmer K, Christiansen JA, Cornel AJ, Hammock BD (2005). Development of a HPLC/Tandem-MS method for the analysis of the larvicides methoprene, hydroprene, and kinoprene at trace levels using Diels-Alder derivatization.. J Agric Food Chem.

[49] Tamura K, Peterson D, Peterson N, Stecher G, Nei M (2011). MEGA5: Molecular evolutionary genetics analysis using maximum likelihood, evolutionary distance, and maximum parsimony methods.. Mol Biol Evol.

[50] Munyiri FN, Ishikawa Y (2007). Molecular cloning and developmental expression of the gene encoding juvenile hormone esterase in the yellow-spotted longicorn beetle, *Psacothea hilaris*.. Insect Biochem Mol Biol.

[51] Hinton AC, Hammock BD (2003). Juvenile hormone esterase (JHE) from *Tenebrio molitor*: full-length cDNA sequence, in vitro expression, and characterization of the recombinant protein.. Insect Biochem Mol Biol.

[52] Feng QL, Ladd TR, Tomkins BL, Sundaram M, Sohi SS (1999). Spruce budworm (*Choristoneura fumiferana*) juvenile hormone esterase: hormonal regulation, developmental expression and cDNA cloning.. Mol Cell Endocrinol.

[53] Hanzlik TN, Abdel-Aal YAI, Harshman LG, Hammock BD (1989). Isolation and sequencing of cDNA clones coding for juvenile hormone esterase from *Heliothis virescens*: evidence for a catalytic mechanism of the serine carboxylesterases different from that of the serine proteases.. J Biol Chem.

[54] Teese MG, Campbell PM, Scott C, Gordon KHJ, Southon A (2010). Gene identification and proteomic analysis of the esterases of the cotton bollworm, *Helicoverpa armigera*.. Insect Biochem Mol Biol.

[55] Hinton AC, Hammock BD (2001). Purification of juvenile hormone esterase and molecular cloning of the cDNA from *Manduca sexta*.. Insect Biochem Mol Biol.

[56] Hirai M, Kamimura M, Kikuchi K, Yasukochi Y, Kiuchi M (2002). cDNA cloning and characterization of *Bombyx mori* juvenile hormone esterase: an inducible gene by the imidazole insect growth regulator KK-42.. Insect Biochem Mol Biol.

[57] Mackert A, do Nascimento AM, Bitondi MMG, Hartfelder K, Simoes ZLP (2008). Identification of a juvenile hormone esterase-like gene in the honey bee, *Apis mellifera* L. - expression analysis and functional assays.. Comp Biochem Physiol, Part B: Biochem Mol Biol.

[58] Liu SH, Yangb BJ, Go JH, Yao XM, Zhang YX (2008). Molecular cloning and characterization of a juvenile hormone esterase gene from brown planthopper, *Nilaparvata lugens*.. J Insect Physiol.

