# Supplementary figures and images for: Juvenile Hormone (JH) Esterase of the Mosquito Culex quinquefasciatus Is Not a Target of the JH Analog Insecticide Methoprene
Source: PLoS One. 2011 Dec 9;6(12):e28392. doi: 10.1371/journal.pone.0028392 (PMC3235118; doi:10.1371/journal.pone.0028392)

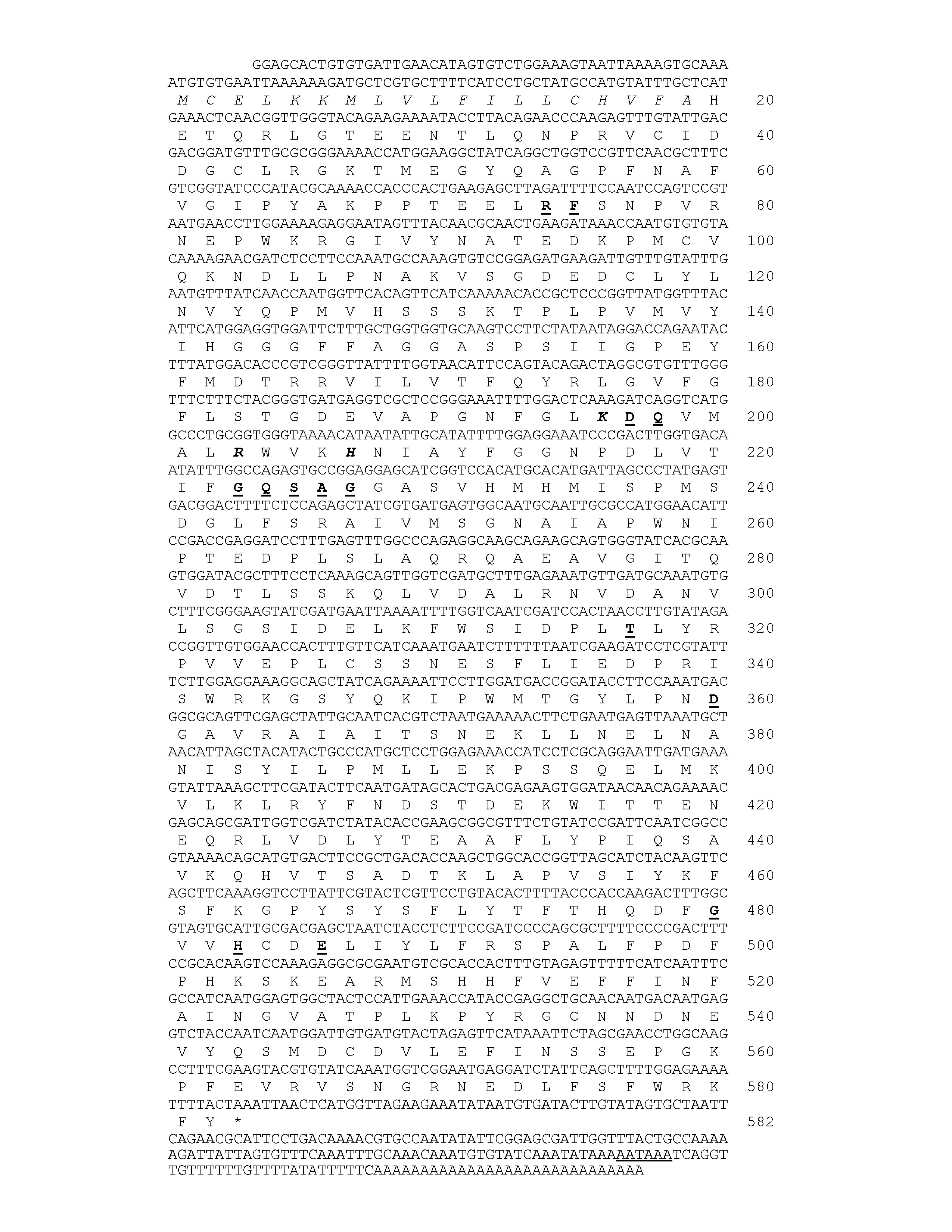

Supplement: Figure S1 — Nucleotide and deduced amino acid sequences of cqjhe and CqJHE. The 5′ and 3′ UTR sequences, and coding sequence of cqjhe were 51, 222, and 1746 nts-long, respectively. Seven amino acid sequence motifs (RF, DQ, GQSAG, E, GxxHxxD/E, R/Kx(6)R/KxxxR, and T) are highly conserved in known JHEs [2], [3]. The RF (residues 74–75), DQ (residues 197–198), GQSAG (residues 223–227), D (residue 360), GxxHxxE (residues 480, 483, and 486), and T (residue 317) motifs are shown in bold underlined text. The E motif (i.e., the acidic amino acid residue of the catalytic triad) is D in CqJHE. The Kx(6)RxxxH motif (residues 196, 203, and 207) is shown in bold italic text. The asterisk indicates a stop codon (TAA). A putative signal peptide sequence (N-terminal 19 amino acid residues) is shown in italic text. A putative CPSF (cleavage and polyadenylation specificity factor) complex binding site is underlined. Amino acid residue positions are indicated to the right. (TIF) [file pone.0028392.s001.tif]

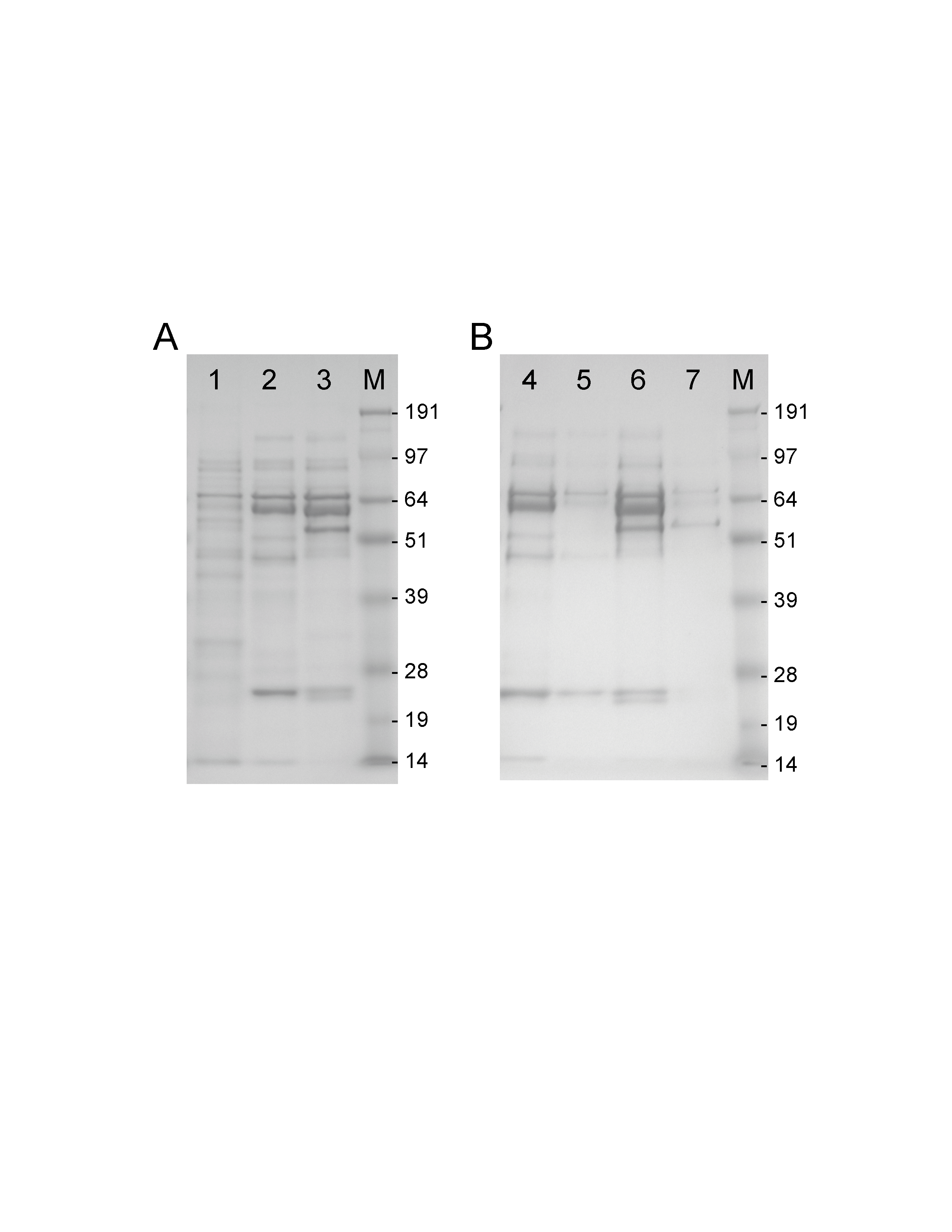

Supplement: Figure S2 — SDS-PAGE analysis of CqJHE following ion exchange purification (A) and binding with MBTFP-Sepharose (B). CqJHE was expressed in High Five cells by AcCqJHE and the culture supernatant (lane 1) was harvested at 65 h postinoculation, diluted (1∶4) with 20 mM Tris-HCl, pH 8.0, and applied onto a strong anion exchange column (Pierce). The column was washed and the proteins were eluted with 20 mM Tris-HCl, pH 8.0, containing increasing concentrations of NaCl. The majority of CqJHE activity eluted in buffer containing 100 (lane 2) or 150 (lane 3) mM NaCl. The protein solutions were desalted and concentrated, and the efficiency of the purification scheme was investigated by treating an equal volume (containing the same amount of total protein) of the 100 mM NaCl (lanes 4 and 5) or 150 mM NaCl (lanes 6 and 7) fractions with MBTFP-Sepharose (lanes 5 and 7), a JHE-selective affinity gel [4]. Treatment of the 100 mM and 150 mM NaCl fractions with MBTFP-Sepharose resulted in 94% and 95% reductions, respectively, in JHE specific activity. The masses (in kDa) of molecular weight standards (lane M) are indicated to the right of each panel. In panel A, 5 µg of total protein was separated in each lane; whereas in panel B, 7.5 µg of total protein was left untreated (lanes 4 and 6) or treated with MBTFP-Sepharose (lanes 5 and 7) prior to separation by SDS-PAGE. (TIF) [file pone.0028392.s002.tif]

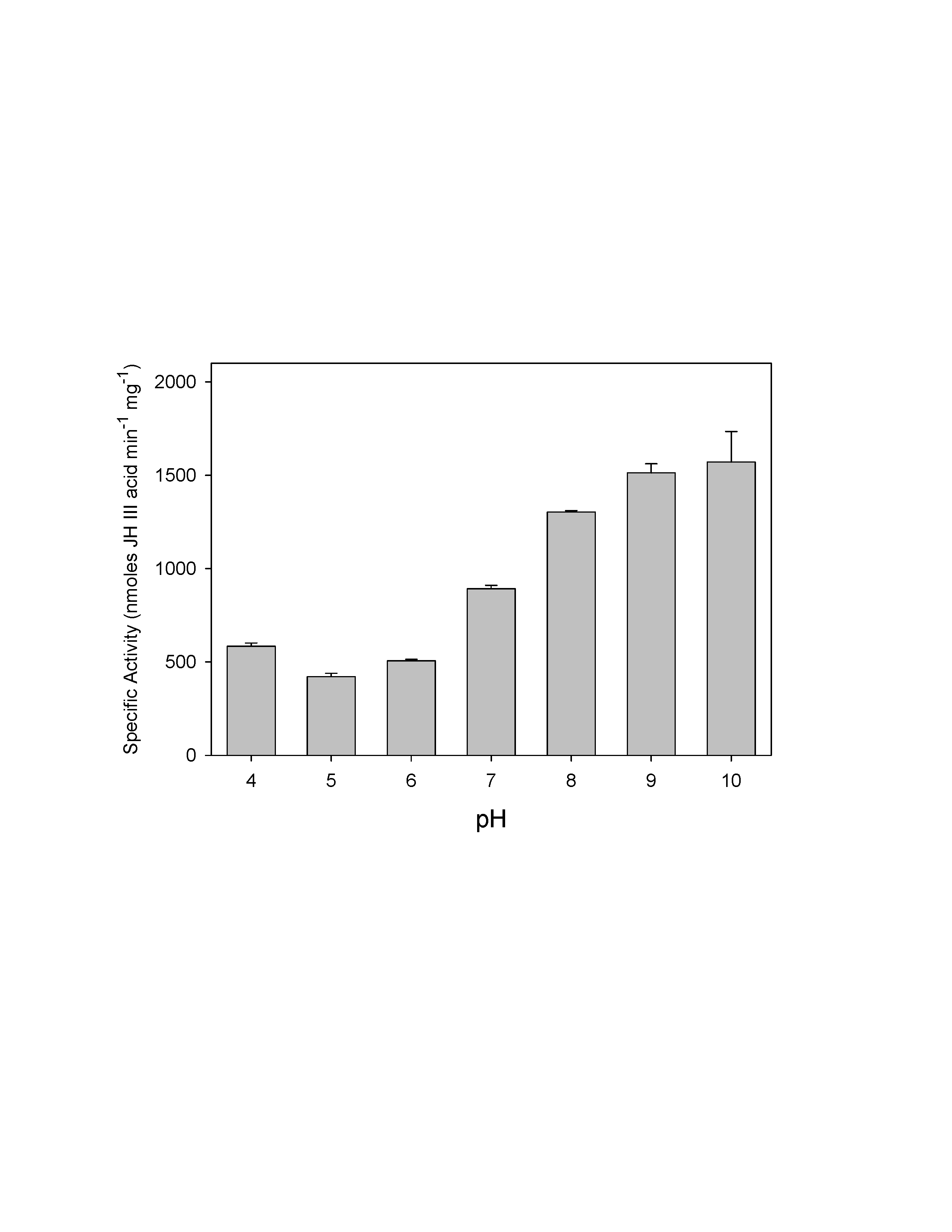

Supplement: Figure S3 — Effect of pH on the specific activity of CqJHE. The partition assay (see Materials and Methods) was performed in citrate-phosphate (pH 4.0 and 5.0), sodium phosphate (pH 6.0, 7.0, and 8.0) or glycine-sodium hydroxide (pH 9.0 and 10.0) buffer containing 0.1 mg ml−1 of BSA. All of the assays were corrected for a low level of background hydrolysis (5.0±3.2%, 2.3±0.4%, 1.4±0.4%, 1.2±0.2%, 1.3±0.3%, 1.4±0.3%, 1.3±0.4% at pH 4, 5, 6, 7, 8, 9, and 10, respectively) that was found at each pH level. The error bars indicate the standard deviation of the mean of at least three independent experiments. (TIF) [file pone.0028392.s003.tif]
