# Supplementary material for: Juvenile Hormone (JH) Esterase of the Mosquito Culex quinquefasciatus Is Not a Target of the JH Analog Insecticide Methoprene
Source: PLoS One. 2011 Dec 9;6(12):e28392. doi: 10.1371/journal.pone.0028392 (PMC3235118; doi:10.1371/journal.pone.0028392)
Supplement: Information S1 — Materials and Methods. (DOC) [file pone.0028392.s004.doc]

Supporting Information:

**Juvenile Hormone (JH) Esterase of the Mosquito *Culex quinquefasciatus* is Not a Target of the JH Analog Insecticide Methoprene**

Shizuo G. Kamita^a,b^, Aman I. Samra^a,b^, Jun-Yan Liu^a,b^, Anthony J. Cornel^a^, and Bruce D. Hammock^a,b,1^

^a^Department of Entomology, University of California, Davis, California, United States of America

^b^UC Davis Cancer Center, Sacramento, California, United States of America

***Supporting Information* Materials and Methods**

**Cloning of the full-length, JHE-encoded cDNA, *cqjhe*, of *Cx. quinquefasciatus*.** Total RNA was isolated from 100 4^th^ instar CQ1 larvae (ca. 500 mg wet weight) using a NucleoSpin L RNA extraction kit (Clontech) following the manufacturer’s protocol. Messenger RNA was isolated from 523 µg of total RNA using a NucleoTrap Midi kit (Clontech) following the manufacturer’s protocol. Double-stranded cDNA (ds cDNA) was generated from 2.91 µg of mRNA (15 cycles of long-distance PCR) using a Creator SMART cDNA Library Construction kit (Clontech) following the manufacturer’s protocol.

The 3’-end and 3’-UTR of the JHE-encoding cDNA of CQ1 (*cqjhe*) were obtained by 3’-RACE PCR using the degenerate primer JHE4 (5’-GTNACNAT(A/C/T)TT(C/T)GGNCA(A/G)AG(C/T)GCNGGN-3’), anchor primer CDSIIIshort (5’-ATTCTAGAGGCCGAGGCGGCCGAC-3’), and CQ1 ds cDNAs as template. The degenerate JHE4 primer was designed on the basis of the amino acid residues VTIFGQSAG that includes the GQSAG motif that is conserved in biologically active JHE proteins. PCR amplification was performed with KOD Hot Start DNA polymerase (Novagen) as follows: 94ºC, 2 min; 25 cycles of 94ºC, 15 sec; 60ºC, 30 sec; 68ºC, 1 min; and one cycle of 68ºC, 5 min. This PCR generated a 0.9 kbp-long amplicon that was gel-purified and inserted into the pCR-Blunt II-TOPO cloning vector (Invitrogen) and transformed into One Shot Top10 competent cells (Invitrogen) following the manufacturer’s protocol. The sequence of the insert was determined (by the UC Davis College of Biological Sciences Sequencing Facility) and confirmed to encode a potential JHE. The 3’-end and 3’-UTR of *CqJHE* were amplified a second time using the gene-specific primer jhe3int3 (5’-CGTTTCTGTATCCGATTCAATCGG-3’), CDSIIIshort primer, and CQ1 ds cDNAs as template. This PCR amplification was performed as described above except with an annealing temperature of 63ºC. The 0.7 kbp-long amplicon was gel-purified, inserted into the pCR-Blunt II-TOPO cloning vector, transformed into One Shot Top10 competent cells, and sequenced as described above.

The 5’-end and 5’-UTR of *cqjhe* were amplified by 5’-RACE PCR using CQ1 ds cDNAs, the anchor primer SMARTshort (5’-AGAGTGGCCATTACGGCCGGG-3’), and gene-specific primer JHE3int (5’-GAAAAGTCCGTCACTCATAGGGC-3’). The PCR amplification was performed as described above except with an annealing temperature of 65ºC. Following the PCR amplification, 1 μl of GoTaq DNA polymerase (Promega) was added to the reaction mixture and incubated for 2 min. The 0.9 kbp-long amplicon was gel-purified, ligated into the cloning vector pCR3.1 (Invitrogen) using a DNA ligation kit 2.1 (Takara), transformed into One Shot Top10 competent cells, and sequenced as described above.

After the 5’- and 3’-ends of *cqjhe* were determined, the predicted JHE-encoding open reading frame (ORF) was amplified by PCR using the primers CQ1JHEfBglII (5’-GGAAGATCTATGTGTGAATTAAAAAAGATGCTC-3’) and CQ1JHErEcoRI-A (5’-CGGAATTCTTAGTAAAATTTTCTCCAAAAGCTGA-3’), and CQ1 ds cDNAs as template. PCR amplification was performed as described above except with an annealing temperature of 65ºC and an elongation time of 2 min for 35 cycles. The 1.7 kbp-long amplicon was gel purified, digested with *Bgl*II then *Eco*RI, and then column purified. The 1.7 kb-long *Bgl*II-*Eco*RI fragment was ligated into the *Bgl*II and *Eco*RI cloning sites of the baculovirus transfer vector pAcUW21 [1] using T4 DNA ligase (New England Biolabs) and transformed into DH5α cells to generate the recombinant transfer vector pAcUW21-CqJHE. The complete sequence of the predicted JHE-encoding ORF in pAcUW21-CqJHE was determined in both directions using pAcUW21-specific and *cqjhe*-specific primers.

**Secretion of CqJHE into the culture medium.** A 19 amino acid residue-long signal peptide for secretion was predicted at the N-terminal of CqJHE. In order to determine whether the recombinant CqJHE expressed by AcCqJHE was secreted into the cell culture medium or retained within the cells, High Five cells (Invitrogen) were inoculated with AcCqJHE at a multiplicity of infection of 0.5 and cultured on ESF921 medium (Expression Systems) at 27ºC. The culture medium and infected cells were harvested at 65 h postinoculation by centrifugation at 2,000 x*g* for 30 min at 4ºC. JH esterase activity in the culture medium (diluted 1,000- to 2,000-fold in 50 mM sodium phosphate, pH 7.4, buffer containing 0.1 mg ml^-1^ of BSA) was determined by a partition assay as described in the Materials and Methods. The cells were washed and resuspended in an equal volume of 50 mM sodium phosphate buffer, pH 7.4, and then homogenized (three 30 sec-long pulses using an Ultra Turrax T-25 (IKEA-Werke) homogenizer followed by passage through a 25 gauge x 5/8” needle). The homogenate was passed through a 0.22 micron filter. JH esterase activity in the filtrate (diluted 10-fold in 50 mM sodium phosphate, pH 7.4, buffer containing 0.1 mg ml^-1^ of BSA) was determined by a partition assay as described in the Materials and Methods.

**Effect of pH on the JH hydrolytic activity of CqJHE.** The JH partition assay was performed using citrate-phosphate (pH 4.0 and 5.0), sodium phosphate (pH 6.0, 7.0, and 8.0) or glycine-sodium hydroxide (pH 9.0 and 10.0) buffer in order to determine the effect of pH on JHE activity. Chemical hydrolysis of JH III was determined at each pH and subtracted as background. All of the assays were performed in triplicate and repeated at least three times.

## *Supporting Information* References

1. Weyer U, Knight S, Possee RD (1990) Analysis of very late gene expression by *Autographa californica* nuclear polyhedrosis virus and the further development of multiple expression vectors. J Gen Virol 71: 1525-1534.

2. Ward VK, Bonning BC, Huang T, Shiotsuki T, Griffeth VN, et al. (1992) Analysis of the catalytic mechanism of juvenile hormone esterase by site-directed mutagenesis. Int J Biochem 24: 1933-1941.

3. Kamita SG, Hammock BD (2010) Juvenile hormone esterase: biochemistry and structure. J Pestic Sci 35: 265-274.

4. Abdel-Aal YAI, Hammock BD (1986) Transition state analogs as ligands for affinity purification of juvenile hormone esterase. Science 233: 1073-1076.
