# Supplementary material for: Juvenile Hormone (JH) Esterase of the Mosquito Culex quinquefasciatus Is Not a Target of the JH Analog Insecticide Methoprene
Source: PLoS One. 2011 Dec 9;6(12):e28392. doi: 10.1371/journal.pone.0028392 (PMC3235118; doi:10.1371/journal.pone.0028392)
Supplement: Table S1 — Purification of CqJHE by ion exchange chromatography. (DOC) [file pone.0028392.s005.doc]

**Table S1. Purification of CqJHE by ion exchange chromatography**

| Fraction | Volume (ml) | Activity (nmoles min^-1^ ml^-1^) | Total Activity (nmoles min^-1^) | Activity Recovered |
| --- | --- | --- | --- | --- |
| High Five supernatant | 90 | 20.9 | 1881 |  |
| loading | 360 | 5.6 | 2016 | 100% |
| flow through | 360 | none detected | none detected | 0 |
| 100 mM NaCl | 48 | 9.1 | 437 | 22% |
| 150 mM NaCl | 48 | 25.1 | 1205 | 60% |
| 200 mM NaCl | 48 | 4.3 | 206 | 10% |
| 300 mM NaCl | 48 | 1.2 | 58 | 3% |
